# Uveitis reactivation following recombinant zoster vaccination

**DOI:** 10.1016/j.ajoc.2021.101115

**Published:** 2021-05-03

**Authors:** Paige J. Richards, Maxwell J. Wingelaar, Karen R. Armbrust, Laura J. Kopplin

**Affiliations:** a University of Wisconsin Department of Ophthalmology and Visual Sciences, United States; b University of Minnesota, Department of Ophthalmology and Visual Neurosciences, United States; c Minneapolis Veterans Affairs Health Care System, Department of Ophthalmology, United States

**Keywords:** Shingrix, Vaccine, Uveitis, Multifocal choroiditis, Autoimmune

## Abstract

**Purpose:**

Describe three cases of uveitis reactivation following immunization with recombinant zoster vaccine (RZV).

**Observations:**

One patient developed reactivation of previously controlled multifocal choroiditis within one week of receiving RZV, requiring treatment with systemic corticosteroids. Two patients with previously controlled anterior uveitis developed new anterior segment inflammation after RZV; both were treated with topical corticosteroids and systemic antiviral therapy.

**Conclusion and importance:**

Uveitis recurrence is an infrequent but serious potential ocular side effect of recombinant zoster vaccination.

## 1 Introduction

Herpes zoster is a viral infection caused by varicella zoster virus (VZV) reactivation. There are two vaccinations available for immunization against herpes zoster: zoster vaccine live (ZVL, Zostavax), a live attenuated vaccine available since 2006, and recombinant zoster vaccine (RZV, Shingrix), a recombinant subunit vaccine available since 2017. The most recent Centers for Disease Control guidelines recommend healthy adults 50 years and older undergo vaccination with RZV, which is administered as a two-dose series with 2–6 months between doses. Post-licensure safety monitoring of RZV by the Vaccine Adverse Event Reporting System found a reporting rate of 0.4/100,000 for inflammatory eye disease, with reported events including herpes zoster keratitis and keratoconjunctivitis, two cases of primary herpes zoster iridocyclitis and one report of pre-existing ophthalmic herpes zoster.^1^ The recombinant zoster vaccine contains a novel adjuvant, AS01_B_, which stimulates a potent immunogenic response that may be responsible for long-lasting cell-mediated immunity.^2^ The increased immunogenicity of the adjuvanted vaccine is one advantage of RZV over ZVL,^3^ but it raises the potential for immune-mediated events, particularly in those with known inflammatory disease. Here we present three cases of patients with reactivation of their previously controlled uveitis after receiving RZV vaccination.

## 2 Findings

### 2.1 Case 1

**Fig. 1:**
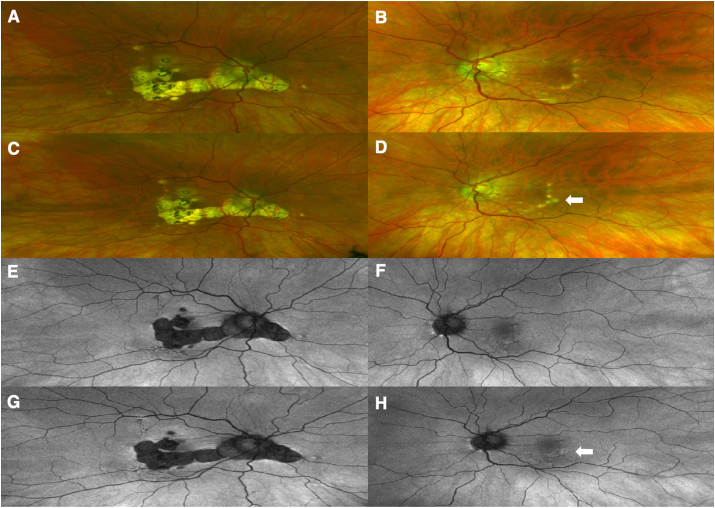
Right (A) and left (B) eye color fundus photographs prior to RZV vaccination demonstrate chorioretinal scarring from prior inflammation. Photos after RZV show stability right eye (C) and a new active inflammatory lesion in the temporal macula of the left eye (D, Arrow). Fundus autofluorescence prior to RZV shows areas of hypoautofluorescence in both eyes (E and F) with small peripapillary hyperautofluorescent spots in the left eye (F). Fundus autofluorescence imaging after RZV demonstrates stability right eye (G) and new hyperautofluorescence at the active lesion in the left eye (H, Arrow). (For interpretation of the references to color in this figure legend, the reader is referred to the Web version of this article.)

**Fig. 2:**
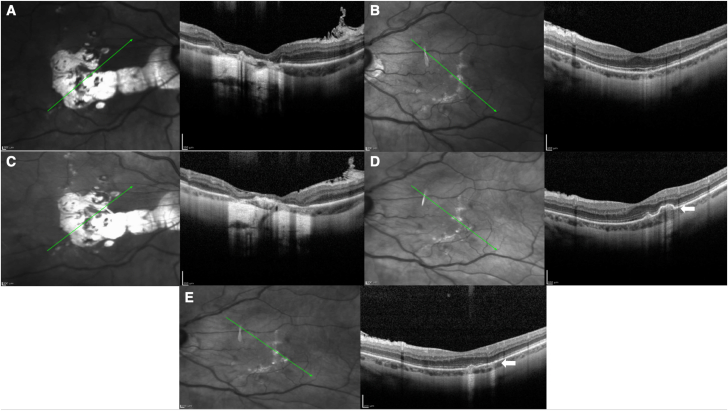
Optical Coherence Tomography (OCT) of the right (A) and left (B) eyes with a transverse cut shows scarring in areas of prior inflammation. OCT taken one week after RZV vaccination (C and D) demonstrates a new chorioretinal lesion in the temporal macula of the left eye (D, Arrow), with subsequent improvement following treatment with oral corticosteroids (E, Arrow).

A 57-year-old Caucasian woman with a history of bilateral multifocal choroiditis controlled on methotrexate 10 mg po weekly presented with an acute decrease in vision in the right eye (OD) and new metamorphopsia in the left eye (OS) five days after receiving her first RZV vaccine. She also reported upper arm swelling at the injection site, chills, malaise, subjective fever, and tinnitus that started 24 hours after the RZV injection. On examination, we measured count fingers vision eccentrically OD (baseline acuity 20/40) and 20/20–2 vision OS with correction. Intraocular pressure was within normal limits in both eyes (OU). Pupils were equal, round, and reactive to light, without evidence of a relative afferent pupillary defect. On slit-lamp exam we noted a quiescent anterior segment OU, an occasional anterior vitreous cell OD, and no vitreous haze in either eye. In the right eye we saw stable posterior segment findings including peripapillary atrophic scarring with temporal thinning of the optic nerve, confluent circular punched-out atrophic macular scars with a small spared foveal region, and vessel attenuation. In the left eye we saw a linear yellow scar temporal to the fovea and a new yellow chorioretinal lesion adjacent to this scar (Fig. 1A–D). By fundus autofluorescence we saw stable hypoautofluorescence in the area of prior retinal scars OD and a new area of hyperautoflurorescence at the site of the new lesion OS (Fig. 1E–H). Despite the decrease in vision OD, there was no change on macular ocular coherence tomography (OCT) compared to previously identified atrophy and scarring. On macular OCT OS we noted a new outer retinal lesion temporal to prior residual scar (Fig. 2A–D). The patient was started on 60 mg of oral prednisone daily and continued methotrexate. On follow-up examination one week later the patient endorsed decreased metamorphopsia in the left eye. Visual acuity was improved to 20/250 OD and remained at 20/20–2 OS. Ocular examination remained stable OD and the new lesion noted at prior examination OS was less elevated (Fig. 2E). The patient underwent a prednisone taper over two months without development of recurrent inflammation; however, she developed a secondary choroidal neovascular membrane at the edge of the new scar requiring treatment with intravitreal bevacizumab.

### 2.2 Case 2

**Fig. 3:**
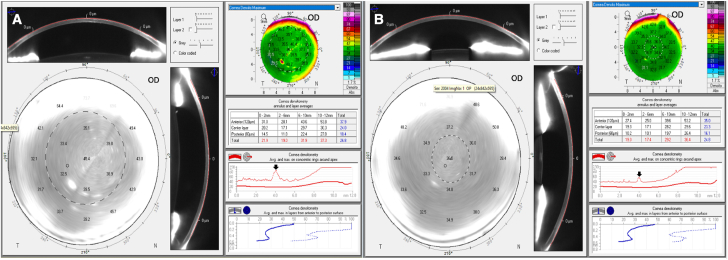
Pentacam corneal densitometry display of the right eye compares corneal light backscatter during active keratitis (A) and following resolution of the keratitis (B). The backscatter values are higher in the central (0–2 mm) and second (2–6 mm) annular zones during active keratitis. The peak in maximal corneal densitometry at the 4 mm annulus during active keratitis shows relative flattening after keratitis resolution (Arrows, Heat Map), consistent with the clinical improvement in corneal clarity.

A 69-year-old male with a history of idiopathic recurrent bilateral anterior and mild intermediate uveitis presented with sudden onset headache and blurred vision in the right eye one month after receiving his second RZV vaccination. He had finished a course of topical prednisolone acetate 1% three months ago OD and two months ago OS, and his uveitis was quiescent on examination two months prior. On exam, his best corrected vision was 20/50 OD and 20/30 in the unaffected OS. Intraocular pressures were within normal limits in both eyes. On exam we noted several foci of anterior stromal keratitis, stellate keratic precipitates and trace anterior chamber cell OD; examination OS was unremarkable. On Pentacam optical densitometry of the cornea, there was loss of clarity in the regions of stromal keratitis (Fig. 3A). The patient was started on valacyclovir 1000 mg three times daily. Three days later the anterior stromal keratitis resolved and there was improvement of anterior chamber cell to 0.5+. Prednisolone acetate 1% drops two times daily OD was initiated for two weeks and the patient completed a two-week course of valacyclovir 1000 mg three times daily followed by 500 mg daily as prophylaxis. At follow up one month after initial presentation his vision improved to 20/30 OD, the stromal keratitis remained resolved, the number of keratic precipitates was reduced and anterior chamber inflammation was quiescent. Corneal densitometry demonstrated improvement (Fig. 3B).

### 2.3 Case 3

A 70-year-old female with a history of recurrent unilateral anterior uveitis and corneal neovascularization with lipid keratopathy OS presented two weeks after receiving her first RZV with mildly decreased vision left eye. She had completed treatment for presumed viral keratouveitis six months prior with a ten-day course of oral valacyclovir 1000 mg three times daily and a tapering course of topical loteprednol etabonate 0.5%. Four months earlier, the patient's uveitis had been quiescent off treatment, but following RZV she developed 1+ anterior chamber cell and new keratic precipitates in the left eye. The patient was treated with oral valacyclovir 1000 mg three times daily followed by 1000 mg daily and topical prednisolone acetate 1% with return to quiescence six weeks later.

## 3 Discussion

The RZV is an adjuvanted subunit vaccine for immunization against herpes zoster. Currently, the Advisory Committee on Immunization Practices recommends RZV vaccination over ZVL in immunocompetent individuals over the age of 50 years. RZV is preferred over the prior live attenuated vaccine since it is more efficacious particularly in older populations, its efficacy is longer lasting, and it may be safely administered to immunocompromised patients for whom a live vaccine is contraindicated.^2^^,^^3^ RZV contains recombinant VZV glycoprotein E and the adjuvant AS01_B_, which is composed of two immunostimulants: a toll-like receptor 4 agonist (3-O-desacyl-40-monophosphoryl lipid) and a saponin derived molecule QS21 (from the South American tree *Quillaja saponaria*). The adjuvant induces an innate immune-cell mediated response, which enhances glycoprotein E antigen presentation to T cells and induces increased production of antibodies and CD4^+^ T cells specific to the VZV glycoprotein E.^2^^,^^3^

Results from two large randomized placebo-controlled phase 3 trials of the RZV found potential immune-mediated diseases occurred at a similar rate between those receiving RZV and controls at all time points. Similarly, subjects with preexisting possible immune-mediated diseases did not demonstrate an increased risk for a new possible immune-mediated process or exacerbation of their prior disease after RZV vaccination compared with controls. Ocular autoimmune diseases were a pre-defined reportable adverse event in both trials; uveitis was only recorded in 1 of 14,645 subjects receiving RZV.^4^ Post-licensure surveillance of RZV in the Vaccine Adverse Event Reporting System found a reporting rate for inflammatory eye disease of 0.4/100,000 with limited reports related to uveitis (two cases of primary herpes zoster iridocyclitis, one report of presumed reactivation of pre-existing ophthalmic herpes zoster without specification of affected ocular structures).^1^ A query of the VAERS database in October 2020, just prior to our submission of the cases included in this report, identified a report of severe unilateral inflammation treated with Kenalog and oral prednisone by a retina specialist; additional details of this case are unknown. The predominant adverse ocular events reported in VAERS include herpes zoster ophthalmicus, keratitis and conjunctivitis; it is unknown whether any of these patients had preexisting inflammatory ocular disease, and the possibility that these cases actually occurred from a lack of vaccine efficiency cannot be excluded.

Review of the literature finds only limited prior cases of uveitis follow RZV vaccination. Heydari-Kamjani et al.^5^ reported a case of presumed subclinical sarcoidosis that subsequently presented with the development of uveitis starting 4 days after vaccination with RZV. Unlike our cases, this patient had no prior history of ocular inflammation. A recent report characterized a case of acute retinal necrosis following RZV.^6^ As RZV does not contain infectious virus, this most likely represented a failure of efficacy in boosting immunity to VZV. There have been two reports of recurrent keratitis after vaccination with RZV. One patient had a history of controlled herpetic stromal keratitis who developed reactivation 3 weeks following the RZV^7^; the other had a remote history of herpes zoster ophthalmicus who presented with stromal keratitis and ulceration a week following receipt of the second RZV dose.^8^

Here, we present a spectrum of cases with uveitis activation in patients with previously controlled ocular inflammation following vaccination with the RZV. Viral DNA from previous zoster infection has been detected in corneal tissue up to eight years following the initial clinical presentation with herpes zoster ophthalmicus.^9^ One possible mechanism for post-RZV ocular inflammation is that the cell-mediated response to RZV vaccination reacts with this residual viral DNA which results in reactivation of viral keratitis or potentially keratouveitis. We propose this as the possible cause for recurrent inflammation in our third case. The mechanism for reactivation of uveitis in our other two cases is less clear. We hypothesize that in case 1, the patient with longstanding multifocal choroiditis who was controlled on immunosuppression, the upregulation of humoral and cell-mediated responses following vaccination may have resulted in reactivation of immune cells directed against uveal antigens in addition to the desired response against the VZV glycoprotein. The uveitis reactivation in case 2 is consistent with a viral process, particularly given the keratitis. The underlying etiology may be a failure of vaccination since the patient's prior uveitis presentation, which was a bilateral process without keratitis that was well controlled with short courses of topical corticosteroids, was not consistent with a viral etiology.

Despite the possibility of uveitis reactivation following RZV, RZV vaccination is an important component of preventative health. The presented cases should not deter patients and physicians from recommending the RZV vaccine; RZV is efficacious in preventing herpes zoster and postherpetic neuralgia. Rather, this report highlights the importance of ensuring primary care providers are aware of a patient's history of immune-mediated eye disease. We recommend that patients with a history of uveitis discuss plans for RZV vaccination with their ophthalmologist and primary care provider in advance, so appropriate postvaccination ocular monitoring occurs.

## 4 Conclusions

Reactivation of uveitis is an uncommon complication of RZV vaccination.

## Financial support

This work was supported in part by an unrestricted grant from 10.13039/100001818Research to Prevent Blindness, and a National Eye Institute Vision Research Core Grant (P30 EY016665) to the 10.13039/100007015University of Wisconsin-Madison Department of Ophthalmology and Visual Sciences.

## Patient consent

Consent to publish the case report was not obtained. This report does not contain any personal information that could lead to the identification of the patient.

## Declaration of competing interest

No conflicting relationships exists for any author.
